# Protective Effects of Influenza Vaccine against Colorectal Cancer in Populations with Chronic Kidney Disease: A Nationwide Population-Based Cohort Study

**DOI:** 10.3390/cancers15082398

**Published:** 2023-04-21

**Authors:** Chun-Chao Chen, Wen-Rui Hao, Hong-Jye Hong, Kuan-Jie Lin, Chun-Chih Chiu, Tsung-Yeh Yang, Yu-Ann Fang, William Jian, Ming-Yao Chen, Min-Huei Hsu, Shih-Chun Lu, Yu-Hsin Lai, Tsung-Lin Yang, Ju-Chi Liu

**Affiliations:** 1 Division of Cardiology, Department of Internal Medicine, Shuang Ho Hospital, Taipei Medical University, New Taipei City 23561, Taiwan; 2 Taipei Heart Institute, Taipei Medical University, Taipei 11031, Taiwan; 3 Division of Cardiology, Department of Internal Medicine, School of Medicine, College of Medicine, Taipei Medical University, Taipei 11031, Taiwan; 4 Graduate Institute of Medical Sciences, College of Medicine, Taipei Medical University, Taipei 110301, Taiwan; 5 School of Chinese Medicine, College of Chinese Medicine, China Medical University, Taichung City 404333, Taiwan; 6 Division of Cardiovascular Surgery, Department of Surgery, Shuang Ho Hospital, Taipei Medical University, New Taipei City 23561, Taiwan; 7 Department of Emergency, University Hospitals Cleveland Medical Center, Cleveland, OH 44106, USA; 8 Division of Gastroenterology and Hepatology, Department of Internal Medicine, School of Medicine, College of Medicine, Taipei Medical University, Taipei 110301, Taiwan; 9 TMU Research Center for Digestive Medicine, Taipei Medical University, Taipei 110301, Taiwan; 10 Division of Gastroenterology and Hepatology, Department of Internal Medicine, Shuang Ho Hospital, New Taipei City 23561, Taiwan; 11 Graduate Institute of Data Science, College of Management, Taipei Medical University, Taipei 110301, Taiwan; 12 Department of Neurosurgery, Shuang Ho Hospital, Taipei Medical University, New Taipei City 23561, Taiwan; 13 Department of Surgery, MacKay Memorial Hospital, Taipei 10449, Taiwan; 14 Division of Cardiology, Department of Internal Medicine and Cardiovascular Research Center, Taipei Medical University Hospital, Taipei 110301, Taiwan

**Keywords:** influenza vaccine, colorectal cancer, chronic kidney disease

## Abstract

**Simple Summary:**

Colorectal cancer, chronic kidney disease, and influenza infection were corelated through the chronic inflammation pathway and dysbiosis in the gut. Influenza infections aggravate the chronic inflammation status and dysbiosis in the gut, which may result in tumorigenesis and cause colorectal cancer. Seasonal influenza vaccination is a health policy in Taiwan. The aim of our study was to investigate whether influenza vaccination had a protective effect against colorectal cancer. A total of 12,985 patients with chronic kidney disease are listed in Taiwan’s National Health Insurance Research Database. The present study demonstrated that the influenza vaccine provides a potential protective effect against colorectal cancer in a population with chronic kidney disease.

**Abstract:**

Chronic kidney disease (CKD) is associated with malignancy, including colorectal cancer, via the potential mechanism of chronic inflammation status. This study aimed to determine whether influenza vaccines can reduce the risk of colorectal cancer in patients with CKD. Our cohort study enrolled 12,985 patients older than 55 years with a diagnosis of CKD in Taiwan from the National Health Insurance Research Database at any time from 1 January 2001 to 31 December 2012. Patients enrolled in the study were divided into a vaccinated and an unvaccinated group. In this study, 7490 and 5495 patients were unvaccinated and vaccinated, respectively. A propensity score was utilized to reduce bias and adjust the results. Cox proportional hazards regression was used to estimate the correlation between the influenza vaccine and colorectal cancer in patients with CKD. The results showed that the influenza vaccine exerted a protective effect against colorectal cancer in populations with CKD. The incidence rate of colon cancer in the vaccinated group was significantly lower than in the unvaccinated group, with an adjusted hazard rate (HR) of 0.38 (95% CI: 0.30–0.48, *p* < 0.05). After the propensity score was adjusted for Charlson comorbidity index, age, sex, dyslipidemia, hypertension, diabetes, monthly income, and level of urbanization, the dose-dependent effect was found, and it revealed adjusted HRs of 0.74 (95% CI: 0.54–1.00, *p* < 0.05), 0.41 (95% CI: 0.30–0.57, *p* < 0.001), 0.16 (95% CI: 0.11–0.25, *p* < 0.001) for one, two to three, and four or more vaccinations, respectively. In summary, the influenza vaccine was found to be associated with a reduced risk of colorectal cancer in CKD patients. This study highlights the potential chemopreventive effect of influenza vaccination among patients with CKD. Future studies are required to determine whether the aforementioned relationship is a causal one.

## 1. Introduction

Chronic kidney disease (CKD) has multiple physiopathology pathways that cause various symptoms affecting the patient’s quality of life. Kidney function may be destroyed as the disease progresses. Approximately 11% of the population in high-income countries has been reported to have CKD. CKD is typically related to anemia, uremic syndrome, and cardiovascular disease; however, reports have revealed patients with CKD have a greater risk of cancer. Furthermore, bowel cancer ranks as the second most prevalent cancer among CKD patients and as the third most frequent cause of cancer-related mortality [1,2].

Patients with CKD are twice as likely to develop colorectal cancer than the general population [3]. Compared with that in the general population, the standardized incidence ratio of colorectal cancer in patients with predialytic CKD was reported to be 1.60 (95% CI: 1.45–1.74) [4]. Individuals in the CKD population not undergoing dialysis have a greater risk of colorectal cancer (hazard ratio, 1.79; 95% confidence interval [CI] 1.41–2.27) [5].

Chronic inflammation status is a symptom of CKD [6] and a key factor linked to various stages of tumor growth [6,7]. Moreover, abnormal metabolic status and uremic toxin retention may alter the gut microbiome in patients with CKD [6,8]. Studies have revealed that the gut microbiome plays a major role in inflammation and tumorigenesis due to the interaction between the bacterium and the host’s immune system [9,10]. Virus-induced chronic inflammation significantly increases the likelihood of cancer development by activating inflammatory signaling pathways [11]. A cross-sectional study reported that the richness and diversity of microbiome communities were significantly lower in patients with influenza H1N1 than in a healthy control group. A positive correlation was also observed between inflammatory cytokines and H1N1-enriched bacteria [12]. Through the suppression of inflammation, the risk of colorectal cancer could be reduced, and colorectal cancer carcinogenesis could be regulated [13]. Previous preclinical studies have indicated that an administrated intratumoral influenza vaccine can increase antitumor CD8+, decrease the level of regulatory B cells within the tumor, increase T cell microenvironment infiltration, increase local IFN-ƴ, and reduce the tumor mass [14,15]. Influenza vaccines also enhance the function of NK cells, promoting tumor cell elimination [14,16,17]. Patients undergoing surgical treatment of a tumor who had received an influenza vaccine had lower overall mortality (HR = 0.89, 95% CI = 0.81–0.99, *p* = 0.03) and cancer-related mortality (HR = 0.82, 95% CI = 0.72–0.94, *p* = 0.007) [18].

Seasonal influenza vaccination is a cost-effective health policy in Taiwan. The influenza vaccine is linked to decreases in the prevalence of chronic inflammation and mortality among postsurgical cancer patients [18]. Our study aimed to explore whether the administration of the influenza vaccine could reduce the occurrence of colorectal cancer in individuals with CKD. To examine the potential protective effects of the influenza vaccine against colorectal cancer in the CKD population in Taiwan, we conducted a cohort study based on population data obtained from Taiwan’s National Health Insurance Research Database (NHIRD) and its reimbursement claims.

## 2. Methods

### 2.1. Data Source

Taiwan established its National Health Insurance (NHI) program in 1995, which has achieved an enrollment rate of around 99% among the country’s 23.7 million residents. Additionally, over 97% of clinics in Taiwan participate in the NHI system. The NHIRD contains comprehensive registration records and files of all enrolled patients, with the data accessed by researchers deidentified to protect patient privacy and prevent the disclosure of specific people or institutions. All diagnoses were documented by physicians based on the International Classification of Diseases, Ninth Revision, Clinical Modification (ICD-9-CM) system [19,20]. The study protocol was approved by both the research committee of the NHIRD and the Taipei Medical University Joint Institutional Review Board (TMU-JIRB No. N201804043).

### 2.2. Participants

**Figure 1:**
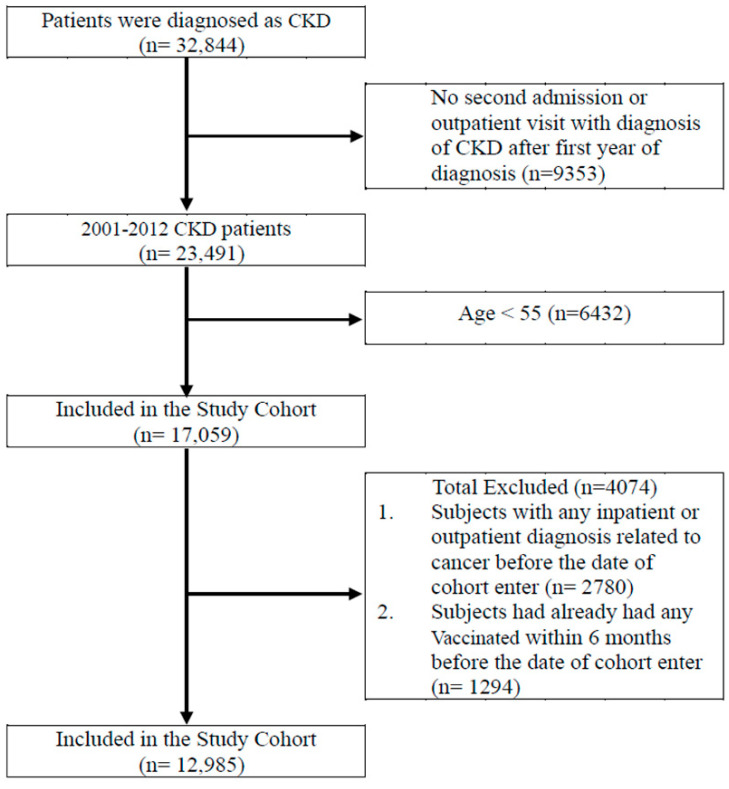
Data selection process.

Our study cohort comprised patients with a diagnosis of CKD, according to the ICD-9-CM, who had visited medical care institutions at any time from 1 January 2001, to 31 December 2012, in Taiwan (*n* = 32,844). We excluded patients without admission records or a second visit within 12 months to the outpatient or emergency department for CKD after the first visit (*n* = 9353). We also excluded patients younger than 55 years old (*n* = 6432) and patients with any inpatient or outpatient record of any malignancy-related disease diagnosed before the enrollment date of our cohort (*n* = 2780). Patients vaccinated in the 6 months before cohort entry (*n* = 1294) were also excluded. Therefore, we enrolled 12,985 patients diagnosed with CKD in Taiwan at any time between 2001 and 2012 in our study (Figure 1).

### 2.3. Potential Confounder

We classified the baseline characteristics and established the comorbidity history for each participant. The characteristics were as follows: age (categorized into four groups: 55–64, 65–74, ≥75 years old), sex (male, female), level of urbanization (urban, suburban, and rural area), monthly income (NTD 0, NTD 1–NTD 21,000, NTD 21,000–NTD 33,300, ≥NTD 33,301 corresponding to USD 0, USD 0.03–USD 700, USD 700–USD 1100, and ≥USD 1100, respectively), Charlson Comorbidity Index (CCI, categorized into four groups: 0, 1, 2, and ≥3) [19,20,21], diabetes (ICD-9-CM code 250), hypertension (ICD-9-CM codes 401–405), and hyperlipidemia (ICD-9-CM code 272). We also recorded the patient’s usage of medication, namely statin, metformin, the renin-angiotensin-aldosterone system [RAA] (such as aldosterone-receptor antagonists, angiotensin-converting enzyme inhibitors, and angiotensin II receptor blockers), and aspirin [22,23,24].

### 2.4. Statical Analysis

Propensity scores (PS) were employed to account for age, gender, Charlson Comorbidity Index (CCI), comorbidities, medication use, monthly income (0, NTD 1 to NTD 21,000, NTD 21,000 to NTD 33,300, and >NTD 33,300), and urbanization level (urban, suburban, and rural) as covariates to achieve balance between the vaccinated and unvaccinated groups and minimize potential bias [25]. Characteristics such as age (55–64, 65–74, and ≥75 years old), gender, comorbidities, medication use, degree of urbanization, and monthly income were compared between the vaccination and comparison cohorts using the chi-squared test. At test was used for comparisons of the continuous variable of mean age. The primary endpoint of our study was the occurrence of colon cancer. In addition to newly diagnosed colon cancer, patients were followed until they dropped out from the NHI program, were lost to follow-up, died, or until 31 December 2012. We used the chi-square test to compare the difference in colon cancer ratios between vaccinated and unvaccinated individuals. The Kaplan–Meier method was used for investigating the colon cancer-free survival rate in patients with CKD who were vaccinated and unvaccinated. Cox proportional hazards regression was employed to determine the hazard ratios (HR) and 95% confidence intervals (CI) to investigate the association between influenza vaccination and the incidence rate of colorectal cancer. A stratified analysis was conducted to assess the effects of vaccination by age and gender. To investigate the dose-dependent effects of influenza vaccination on the incidence of colorectal cancer, we analyzed four patient categories: those who did not receive vaccination and those who received one, two to three, or more than four rounds of vaccination. The total number of vaccinations is defined by the total number of vaccinations a patient received from the index day to primary end point. We conducted a sensitivity analysis to improve our understanding of the effects of covariates on outcomes [20]. Therefore, we analyzed the associations of age; sex; CCI score; diabetes; hypertension; dyslipidemia; and the usage of a statin, metformin, RAA, and aspirin with colorectal cancer incidence in various models in the sensitivity analysis. All statistical analyses were conducted using SPSS Version 22.0 (SPSS, Chicago, IL, USA) and SAS 9.4 software (SAS Institute, Cary, NC, USA). The significance criterion was set at *p* < 0.05.

## 3. Result

### 3.1. Baseline Characteristics among Vaccinated and Unvaccinated Groups

A total of 12,985 individuals diagnosed with CKD between 2001 and 2012 enrolled in our cohort, with 5495 influenza-vaccinated (42.3%) and 7490 unvaccinated people (57.7%). The vaccinated and unvaccinated groups significantly differed in their age distribution, CCI index, level of urbanization, and monthly income. Vaccinated individuals tended to be older than unvaccinated individuals (Table 1). The patients in the unvaccinated group lived in urban areas more than the vaccinated group. We found significant differences between the vaccinated and unvaccinated groups in the prevalence of hypertension, dyslipidemia, and diabetes, and the medical usage of statin, metformin, aspirin, and RAA. The patients in the vaccinated group had taken chronic disease medication longer than those in the unvaccinated group. The total follow-up periods for the unvaccinated and vaccinated groups were 21,919.2 and 33,990.2 person-years, respectively.

### 3.2. Age and Sex among Vaccinated and Unvaccinated Groups

**Figure 2:**
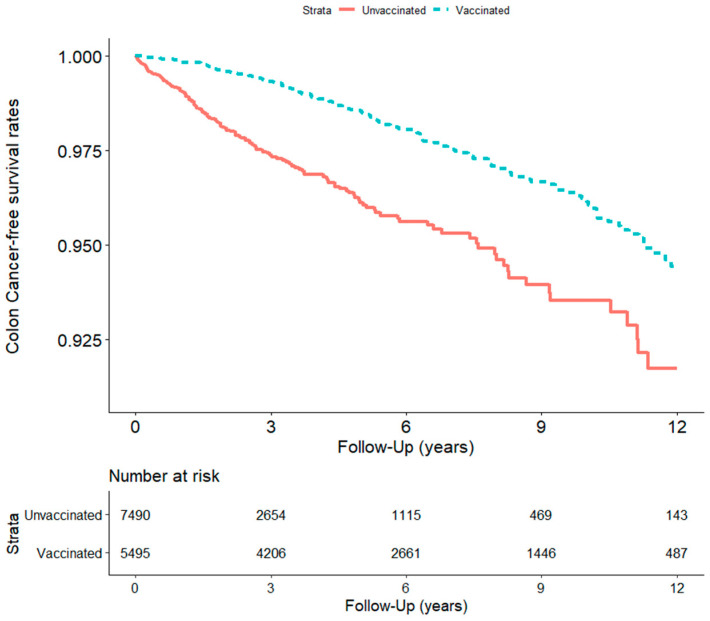
Free of colon cancer survival rate in vaccinated and unvaccinated group.

The incidence rate of colon cancer in the vaccinated group was significantly lower than in the unvaccinated group, with an adjusted HR of 0.38 (95% CI: 0.30–0.48, *p* < 0.05) (Table 2 and Figure 2). In a subgroup analysis by age, the vaccinated group had a lower incidence rate of colon cancer at the ages of 65–74 and older than 75 years, with HRs of 0.31 (95% CI: 0.22–0.44, *p* < 0.05) and 0.35 (95% CI: 0.23–0.52, *p* < 0.05), respectively. Similar effects were seen in both sexes, with HRs of 0.34 (95% CI: 0.22–0.52, *p* < 0.05) and 0.40 (95% CI: 0.30–0.53, *p* < 0.05) in women and men, respectively. The incidence rate of colorectal cancer in both groups increased with age. The incidence rates of colorectal cancer in the unvaccinated group at the ages of 55–64, 65–74, and >75 years were 361.5 (95% CI: 248.1–475.0), 1222.7 (95% CI: 945.9–1499.4), and 1220.6 (95% CI: 914.3–1526.9) per 10^5^ person-years, respectively. However, the incidence rates were 277.8 (95% CI: 168.9–386.8), 391.9 (95% CI: 294.3–489.4), and 447.1 (95% CI: 310.2–584.0) per 10^5^ person-years in the vaccinated group at ages 55–64, 65–74, and >75 years, respectively. Women had a lower incidence rate than men in both the vaccinated and unvaccinated groups. The incidence rates were 643.4 (95% CI: 483.2–803.5) and 247.0 (95% CI:167.4–326.6) for women in the unvaccinated and vaccinated groups, respectively. The incidence rates were 920.0 (95% CI:750.4–1089.6) and 478.7 (95% CI:380.3–577.0) for men in the unvaccinated and vaccinated groups, respectively.

### 3.3. Sensitivity Analysis

**Figure 3:**
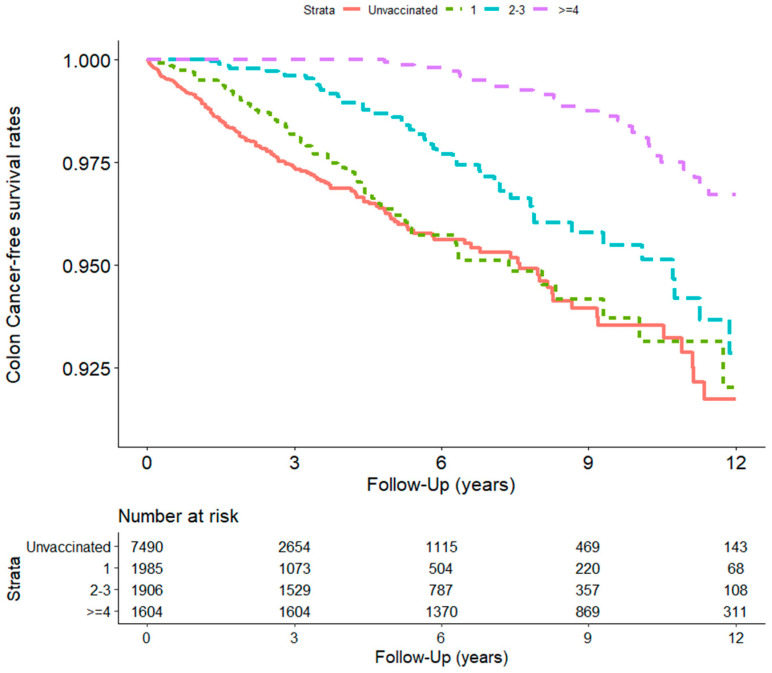
Free of colon cancer survival rate in different numbers of vaccination.

Multiple covariates (age, sex, comorbidity, urbanization level, socioeconomic factor) had their PSs adjusted for in a sensitivity analysis. The influenza vaccine exerted a significant protective effect in the main model and subgroups of covariates (Table 3). The dose-dependent effect of a vaccine was further determined in our study. The adjusted HRs of the main model were 0.74 (95% CI: 0.54–1.00, *p* < 0.05), 0.41 (95% CI: 0.30–0.57, *p* < 0.001), 0.16 (95% CI: 0.11–0.25, *p* < 0.001), for one, two to three, and four or more rounds of vaccination, respectively. The administration of more than two vaccination shots provided a strong protective effect against colorectal cancer. At younger ages (55–64 years old), patients had to receive more than four shots of vaccination for the protective effect of the influenza vaccine to become significant. The adjusted HRs of age for 55–64 years old were 0.86 (95% CI: 0.43–1.74), 0.76 (95% CI: 0.36–1.57), 0.39 (95% CI: 0.16–0.94, *p* < 0.05) for one, two to three, and four or more rounds of vaccination, respectively. However, in the older age group (≥65 years old), the administration of two or more vaccination shots could significantly protect against colorectal cancer. The adjusted HRs for age from 65–74 years old were 0.71 (95% CI: 0.45–1.11), 0.36 (95% CI: 0.22–0.58, *p* < 0.001), and 0.12 (95% CI: 0.07–0.22, *p* < 0.001) for one, two to three, and four or more rounds of vaccination, respectively. The adjusted HRs for those older than 75 years were 0.63 (95% CI: 0.38–1.06), 0.31 (95% CI: 0.17–0.55, *p* < 0.001), and 0.17 (95% CI: 0.08–0.37, *p* < 0.001) for one, two to three, and ≥ four vaccinations, respectively. In the subgroup of CCI ≥ 3, even one shot of influenza vaccination could protect the patient against colorectal cancer. The adjusted HRs for CCI ≥ 3 were 0.55 (95% CI: 0.35–0.84, *p* < 0.01), 0.30 (95% CI: 0.18–0.48, *p* < 0.001), and 0.08 (95% CI: 0.03–0.17, *p* < 0.001), for one, two to three, and four or more rounds of vaccination, respectively (Table 3, Figure 3).

## 4. Discussion

This is the first study to demonstrate the protective effect of the influenza vaccine against colorectal cancer in patients with CKD. In the present study, we found that the incidence rate of colorectal cancer decreased by approximately 50% for those with CKD who received the influenza vaccination. The dose-dependent effect indicated that patients who received more shots of the influenza vaccine might acquire a stronger protective effect. In the younger age subgroup (55–64 years old), the patient may receive four or more shots of the influenza vaccine to acquire protective effects against colorectal cancer. The protective effect is superior in patients with a CCI ≥ 3.

Studies describing the association between the influenza vaccine and colorectal cancer have indicated that receiving an influenza vaccine 6–12 months before colorectal surgery may reduce the risk of tumor recurrence (HR: 0.78, 95% CI: 0.67–0.90) [14]. Another study revealed that receiving an influenza vaccine 0–30 days after the surgery of a solid tumor could reduce the incidence rate of overall (HR: 0.73, 95% CI: 0.60–0.89, *p* = 0.002) and cancer-related mortality (HR: 0.70, 95% CI: 0.53–0.91, *p* = 0.009) [18].

Several mechanisms can be proposed as explanations for our results. First, multiple factors resulting in dysbiosis may be at work among patients with CKD; these factors include uremic toxin retention, metabolic acidosis, and fluid overload with intestinal wall congestion. Drug intake (antibiotics, iron supplements, and polymer phosphate binders) and diet (decreased fiber intake) are also critical factors that change the composition of the microbiome community in the gut [26]. Metabolic changes in patients with CKD may upset the balance of symbionts and pathobionts; therefore, they promote the overgrowth of pathobionts and induce inflammation and the loss of intestinal barrier function. Second, innate immunity was activated, and proinflammatory cytokines were produced due to metabolic changes [27]. The impaired intestinal barrier resulted in increased intestinal colonic permeability and an abnormal colonic epithelial barrier (leaky gut) [8]. In this situation, bacteria may activate myeloid differentiation factor 88 (MyD88) by engaging toll-like receptors on tumor-infiltrating myeloid cells and increasing the production of interleukin (IL)-23. IL-23 could activate IL-17A, IL-6, IL-22, and nuclear factor-κB (NFκB) with a signal transducer and the transcription-3 (STAT3) pathway. These reactions would eventually lead to tumor cell proliferation [28,29,30]. The increase in IL-17C caused by commensal bacteria would induce the expression of B-cell lymphoma-2 and Bcl-xL in intestinal epithelial cells, leading to tumor cell survival and tumorigenesis [30].

Besides, bacterial overgrowth and advanced antibiotic usage may lead to tumorigenesis. A study reported that antibiotics such as aminoglycoside, beta-lactam, and quinolones may affect mitochondrial function, which may result in DNA damage by reactive oxygen species (ROS) produced from mitochondria [31]. Moreover, not only antibiotics but also bacteria could produce ROS directly [10]. The bacteria may release various substances, such as exotoxin and lipopolysaccharide. These molecules resulted in the production of ROS, which caused DNA damage, tumorigenesis, and the inactivation of tumor suppressors [10,31].

However, a study revealed that influenza infection could also affect the microbiome in the gut and lead to dysbiosis. Influenza lung infection may change the composition of the microbiome in the gut due to type 1 interferons produced in the respiratory tract [12,32]. Furthermore, the intestine would be injured while the lung was infected by influenza. Lung-derived CCR9 + CD4 + T cells may migrate to the intestine and increase the production of IFN-γ [33]. Additionally, the reduced amount of butyrate-producing bacteria affected by influenza may cause an overgrowth of pathogenic bacteria. The microbiome alteration may result in a leaky gut and elevated endotoxin concentrations in the blood. Finally, the inflammatory status would be further activated, exacerbating cytokine release [34,35].

This mechanism may explain why the CKD population has a higher incidence rate of colorectal cancer [3,4]. The influenza vaccine may be a convenient and cost-effective method to protect individuals with CKD. However, the dose-dependent effect in our cohort indicated that in younger populations or populations with a lower CCI index, more shots of influenza vaccination may be required to achieve a strong protective effect.

The safety of influenza vaccine injection among patients with cancer receiving immune checkpoint inhibitors, immunotherapy, or chemotherapy has been reported. A meta-analysis demonstrated that the mortality rate (OR = 1.25, 95% CI = 0.81–1.92), p = non-significant) and the incidence of immune-related adverse events (OR = 0.82, 95% CI = 0.63–1.08, p = non-significant) in cancer patients receiving immune checkpoint inhibitors with influenza vaccination had no significant outcome compared to nonvaccinated patients [36].

Another study reported that applying the influenza vaccine to cancer patients receiving immunotherapy had better overall survival if the patient received the flu vaccine and/or developed influenza syndrome (HR = 0.71, 95%CI = 0.50–0.99, *p* = 0.044) [37].

In the colorectal cancer population with active chemotherapy, influenza vaccination also provides enough serological immune response. A study showed no difference in the serological response to the influenza vaccine between active chemotherapy patients and the patients on surveillance (OR = 0.78, *p* = 0.8) [38].

Although our study showed that the influenza vaccine provided a potential protective effect against colorectal cancer in the CKD population, not like the human papillomavirus vaccine, which could block the tumorigenesis pathway directly, the actual mechanisms and pathways may need more research and evidence to prove the causal relationship between the vaccine and the disease. Further research and clinical trials need to be conducted in the future for more solid evidence and pathophysiology mechanisms.

Our study had some limitations. First, our data were all electronic; therefore, coding errors, a common problem in NHIRD cohorts, could not be avoided entirely. In response, we set stricter criteria to improve the accuracy of the diagnosis. The patients enrolled in our study had to be recorded twice for CKD ICD-9CM within 12 months of their first admission or outpatient electronic profile. Second, we could not obtain data on several confounding factors from the NHIRD database, such as diet, body mass index, alcohol consumption, smoking status, and estimated glomerular filtration rate. Thus, we used the PS method to weaken the influence of bias and applied it in the nonrandomized control group to adjust the intervention results between the intervention and control groups [25,39]. Third, the influenza vaccination policy in Taiwan is a form of social welfare for older residents. Therefore, we only enrolled patients older than 55 years in our study. Future studies must include younger patients. Fourth, the observational study tends to make the outcome more effective, thus the PS method was used to reduce the potential bias and make the result more reliable. Finally, our retrospective cohort study design precluded causal inference; thus, future clinical trials should be conducted.

## 5. Conclusions

Influenza vaccination is associated with a lower risk of colorectal cancer among patients with CKD. Sensitivity analysis with multiple covariates revealed a dose-dependent effect of the vaccine in our study. Our study highlights the potential benefits of influenza vaccination in reducing the risk of colorectal cancer in high-risk populations with CKD. Further clinical trials should be conducted to provide more solid evidence and to identify the underlying pathophysiology mechanisms.

## Figures and Tables

**Table 1:** Characteristics of sample population.

	Whole Cohort (*n* = 12,985)		Unvaccinated (*n* = 7490)		Vaccinated (*n* = 5495)		*p* ^a^
	*n*	%	*n*	%	*n*	%	
Age, years (mean ± SD)	70.98 (9.40)		70.09 (10.26)		72.18 (7.90)		<0.001
55–64	3989	30.72	2877	38.41	1112	20.24	<0.001
65–74	4541	34.97	2139	28.56	2402	43.71	
≥75	4455	34.31	2474	33.03	1981	36.05	
Gender							
Female	5712	43.99	3333	44.50	2379	43.29	0.172
Male	7273	56.01	4157	55.50	3116	56.71	
CCI Index ^+^							
0	1491	11.48	876	11.70	615	11.19	0.013
1	2043	15.73	1166	15.57	877	15.96	
2	2876	22.15	1589	21.21	1287	23.42	
≥3	6575	50.64	3859	51.52	2716	49.43	
Diabetes							
No	6310	48.59	3355	44.79	2955	53.78	<0.001
Yes	6675	51.41	4135	55.21	2540	46.22	
Hypertension							
No	2555	19.68	1387	18.52	1168	21.26	<0.001
Yes	10,430	80.32	6103	81.48	4327	78.74	
Dyslipidemia							
No	6337	48.80	3386	45.21	2951	53.70	<0.001
Yes	6648	51.20	4104	54.79	2544	46.30	
Statin							
<28 days	7972	61.39	4786	63.90	3186	57.98	<0.001
28–365 days	2683	20.66	1576	21.04	1107	20.15	
>365 days	2330	17.94	1128	15.06	1202	21.87	
Metformin							
<28 days	10,266	79.06	6045	80.71	4221	76.82	<0.001
28–365 days	1331	10.25	804	10.73	527	9.59	
>365 days	1388	10.69	641	8.56	747	13.59	
RAA							
<28 days	4114	31.68	2792	37.28	1322	24.06	<0.001
28–365 days	3751	28.89	2344	31.30	1407	25.61	
>365 days	5120	39.43	2354	31.43	2766	50.34	
Aspirin							
<28 days	6715	51.71	4478	59.79	2237	40.71	<0.001
28–365 days	3149	24.25	1702	22.72	1447	26.33	
>365 days	3121	24.04	1310	17.49	1811	32.96	
Level of urbanization							
Urban	8785	67.65	5350	71.43	3435	62.51	<0.001
Suburban	2806	21.61	1488	19.87	1318	23.99	
Rural	1394	10.74	652	8.70	742	13.50	
Monthly income (NTD)							
0	1596	12.29	901	12.03	695	12.65	<0.001
1–21,000	4486	34.55	2397	32.00	2089	38.02	
21,000–33,300	3788	29.17	1996	26.65	1792	32.61	
≥33,301	3115	23.99	2196	29.32	919	16.72	

^a^ Comparison between unvaccinated and vaccinated. ^+^ CCI Index: Charlson Comorbidity Index. SD: standard deviation. RAA: renin-angiotensin-aldosterone system. NTD: New Taiwan Dollar.

**Table 2:** Risk of colon cancer among unvaccinated and vaccinated in study cohort.

All Group (*n* = 12,985)	Unvaccinated (Total Follow-up 21,919.2 Person-Years)				Vaccinated (Total Follow-up 33,990.2 Person-Years)				*χ* ^2^	Adjusted HR ^†^ (95% C.I.)
	No. of Patients with Cancer	Incidence Rate (Per 10^5^ Person-Years) (95% C.I.)			No. of Patients With Cancer	Incidence Rate (Per 10^5^ Person-Years) (95% C.I.)				
	175	798.4	(680.1,	916.7)	128	376.6	(311.3,	441.8)	0.001	0.38 (0.30, 0.48) ***
Age, 55–64 ^a^	39	361.5	(248.1,	475.0)	25	277.8	(168.9,	386.8)	3.502	0.65 (0.39, 1.09)
Age, 65–74 ^b^	75	1222.7	(945.9,	1499.4)	62	391.9	(294.3,	489.4)	3.001	0.31 (0.22, 0.44) ***
Age, ≥75 ^c^	61	1220.6	(914.3,	1526.9)	41	447.1	(310.2,	584.0)	0.604	0.35 (0.23, 0.52) ***
Female ^d^	62	643.4	(483.2,	803.5)	37	247.0	(167.4,	326.6)	0.589	0.34 (0.22, 0.52) ***
Male ^e^	113	920.0	(750.4,	1089.6)	91	478.7	(380.3,	577.0)	0.198	0.40 (0.30, 0.53) ***

^a^ Total follow-up person-years of 10,787.5 for unvaccinated and 8998.0 for vaccinated individuals. ^b^ Total follow-up person-years of 6134.2 for unvaccinated and 15,821.9 for vaccinated individuals. ^c^ Total follow-up person-years of 4997.5 for unvaccinated and 9170.3 for vaccinated individuals. ^d^ Total follow-up person-years of 9636.6 for unvaccinated and 14,979.9 for vaccinated individuals. ^e^ Total follow-up person-years of 12,282.6 for unvaccinated and 19,010.3 for vaccinated individuals. C.I.: confidence interval. HR: hazard ratio. † Main model is adjusted for age, sex, Charlson Comorbidity Index, diabetes, hypertension, dyslipidemia, level of urbanization, and monthly income in propensity score. ***: *p* < 0.001. χ^2^: chi-square test statistic

**Table 3:** Sensitivity analysis of adjusted HRs of vaccination in risk reduction of colon cancer.

	Unvaccinated	Vaccinated			*p* for Trend
		1	2–3	≥4	
	Adjusted HR (95% C.I.)	Adjusted HR (95% C.I.)	Adjusted HR (95% C.I.)	Adjusted HR (95% C.I.)	
Main model ^†^	1.00	0.74 (0.54, 1.00) *	0.41 (0.30, 0.57) ***	0.16 (0.11, 0.25) ***	<0.001
Additional covariates ^‡^					
Main model + statin	1.00	0.75 (0.55, 1.01)	0.42 (0.30, 0.59) ***	0.17 (0.11, 0.26) ***	<0.001
Main model + metformin	1.00	0.74 (0.55, 1.01)	0.42 (0.30, 0.58) ***	0.17 (0.11, 0.25) ***	<0.001
Main model + RAA	1.00	0.76 (0.56, 1.04)	0.43 (0.31, 0.60) ***	0.18 (0.12, 0.27) ***	<0.001
Main model + aspirin	1.00	0.77 (0.57, 1.05)	0.44 (0.32, 0.62) ***	0.18 (0.12, 0.28) ***	<0.001
Subgroup effects					
Age, years					
55–64	1.00	0.86 (0.43, 1.74)	0.76 (0.36, 1.57)	0.39 (0.16, 0.94) *	0.037
65–74	1.00	0.71 (0.45, 1.11)	0.36 (0.22, 0.58) ***	0.12 (0.07, 0.22) ***	<0.001
≥75	1.00	0.63 (0.38, 1.06)	0.31 (0.17, 0.55) ***	0.17 (0.08, 0.37) ***	<0.001
Sex					
Female	1.00	0.57 (0.32, 1.01)	0.44 (0.25, 0.77) **	0.11 (0.05, 0.27) ***	<0.001
Male	1.00	0.84 (0.58, 1.21)	0.40 (0.26, 0.60) ***	0.19 (0.11, 0.30) ***	<0.001
CCI Index ^+^					
0	1.00	0.66 (0.23, 1.94)	0.80 (0.34, 1.84)	0.07 (0.01, 0.49) **	0.004
1	1.00	0.75 (0.31, 1.80)	0.26 (0.08, 0.90) *	0.31 (0.12, 0.79) *	0.005
2	1.00	1.23 (0.70, 2.17)	0.57 (0.30, 1.09)	0.30 (0.16, 0.59) ***	<0.001
≥3	1.00	0.55 (0.35, 0.84)**	0.30 (0.18, 0.48) ***	0.08 (0.03, 0.17) ***	<0.001
Diabetes					
No	1.00	0.67 (0.44, 1.02)	0.38 (0.24, 0.59) ***	0.19 (0.12, 0.31) ***	<0.001
Yes	1.00	0.80 (0.51, 1.25)	0.44 (0.27, 0.73) **	0.10 (0.04, 0.25) ***	<0.001
Dyslipidemia					
No	1.00	0.72 (0.48, 1.09)	0.47 (0.31, 0.72) ***	0.22 (0.14, 0.36) ***	<0.001
Yes	1.00	0.74 (0.47, 1.16)	0.31 (0.18, 0.54) ***	0.07 (0.03, 0.18) ***	<0.001
Hypertension					
No	1.00	0.61 (0.32, 1.14)	0.49 (0.27, 0.89) *	0.22 (0.11, 0.43) ***	<0.001
Yes	1.00	0.78 (0.55, 1.10)	0.38 (0.25, 0.56) ***	0.14 (0.08, 0.23) ***	<0.001
Statin					
<28 days	1.00	0.67 (0.46, 0.98) *	0.41 (0.27, 0.62) ***	0.17 (0.10, 0.29) ***	<0.001
28–365 days	1.00	0.95 (0.51, 1.76)	0.28 (0.12, 0.64) **	0.20 (0.08, 0.46) ***	<0.001
>365 days	1.00	0.89 (0.34, 2.32)	0.84 (0.36, 1.98)	0.15 (0.04, 0.54) **	0.004
Metformin					
<28 days	1.00	0.73 (0.52, 1.02)	0.40 (0.28, 0.58) ***	0.15 (0.09, 0.24) ***	<0.001
28–365 days	1.00	1.30 (0.44, 3.84)	0.71 (0.21, 2.47)	0.34 (0.07, 1.72)	0.168
>365 days	1.00	0.61 (0.22, 1.64)	0.37 (0.15, 0.96) *	0.21 (0.08, 0.56) **	0.001
RAA					
<28 days	1.00	0.39 (0.20, 0.76) **	0.37 (0.20, 0.68) **	0.22 (0.11, 0.44) ***	<0.001
28–365 days	1.00	0.91 (0.53, 1.58)	0.34 (0.17, 0.68) **	0.14 (0.06, 0.36) ***	<0.001
>365 days	1.00	1.09 (0.67, 1.75)	0.59 (0.35, 0.98) *	0.19 (0.10, 0.35) ***	<0.001
Aspirin					
<28 days	1.00	0.61 (0.39, 0.94) *	0.31 (0.18, 0.53) ***	0.15 (0.08, 0.29) ***	<0.001
28–365 days	1.00	0.95 (0.55, 1.67)	0.60 (0.34, 1.06)	0.12 (0.05, 0.31) ***	<0.001
>365 days	1.00	1.29 (0.61, 2.70)	0.69 (0.32, 1.48)	0.39 (0.19, 0.83) *	<0.006

* *p* < 0.05 **; *p* < 0.01 ***; *p* < 0.001. HR: hazard ratio. ^+^ CCI Index: Charlson Comorbidity Index. ^†^ Main model is adjusted for age, sex, Charlson Comorbidity Index, diabetes, hypertension, dyslipidemia, level of urbanization, and monthly income in propensity score. ^‡^ The models were adjusted for covariates in the main model and each additionally listed covariate.

## Data Availability

The data supporting the findings of this research were sourced from NHIRD in Taiwan. Owing to the legal restrictions imposed by the Government of Taiwan related to the Personal Information Protection Act, the database cannot be made publicly available.
